# Different types of physical activity are positively associated with indicators of mental health and psychological wellbeing in rheumatoid arthritis during COVID-19

**DOI:** 10.1007/s00296-020-04751-w

**Published:** 2020-11-30

**Authors:** Sophia M. Brady, Sally A. M. Fenton, George S. Metsios, Ailsa Bosworth, Joan L. Duda, George D. Kitas, Jet J. C. S. Veldhuijzen van Zanten

**Affiliations:** 1 School of Sport, Exercise and Rehabilitation Sciences, University of Birmingham, Birmingham, B15 2TT UK; 2 Rheumatology Department, Dudley Group NHS Foundation Trust, Dudley, UK; 3 Medical Research Council-Versus Arthritis Centre for Musculoskeletal Ageing, University of Birmingham, Birmingham, UK; 4 Faculty of Education, Health and Wellbeing, University of Wolverhampton, Wolverhampton, UK; 5 National Rheumatoid Arthritis Society, Maidenhead, UK

**Keywords:** Physical activity, Sedentary behaviour, Mental health, COVID-19, Self-isolation, Rheumatoid arthritis

## Abstract

Nationwide lockdowns during SARS-CoV-2 (COVID-19) can compromise mental health and psychological wellbeing and limit opportunities for physical activity (PA), particularly in clinical populations, such as people with rheumatoid arthritis (RA), who are considered at risk for COVID-19 complications. This study aimed to investigate associations between PA and sedentary time (ST) with indicators of mental health and wellbeing in RA during COVID-19 lockdown, and examine the moderation effects of self-isolating. 345 RA patients completed an online questionnaire measuring PA (NIH-AARP Diet and Health Study Questionnaire), ST (International Physical Activity Questionnaire-Short Form), pain (McGill Pain Questionnaire and Visual Analogue Scale), fatigue (Multidimensional Fatigue Inventory), depressive and anxious symptoms (Hospital Anxiety and Depression Scale), and vitality (Subjective Vitality Scale) during the United Kingdom COVID-19 lockdown. Associations between PA and ST with mental health and wellbeing were examined using hierarchical multiple linear regressions. Light PA (LPA) was significantly negatively associated with mental fatigue (*β* = − .11), depressive symptoms (*β* = − .14), and positively with vitality (*β* = .13). Walking was negatively related to physical fatigue (β = − .11) and depressive symptoms (*β* = − .12) and positively with vitality (*β* = .15). Exercise was negatively associated with physical (*β* = − .19) and general (*β* = − .12) fatigue and depressive symptoms (*β* = − .09). ST was positively associated with physical fatigue (*β* = .19). Moderation analyses showed that LPA was related to lower mental fatigue and better vitality in people not self-isolating, and walking with lower physical fatigue in people self-isolating. These findings show the importance of encouraging PA for people with RA during a lockdown period for mental health and wellbeing.

**Electronic supplementary material:**

The online version of this article (10.1007/s00296-020-04751-w) contains supplementary material, which is available to authorized users.

## Introduction

SARS-CoV-2 (COVID-19) has been declared a pandemic by the World Health Organisation (WHO) [1]. Unprecedented nationwide restrictions were put in place to limit the spread of the virus. In the United Kingdom (UK), the general population was instructed to only leave their home for basic necessities (i.e. food shopping and medical treatment), essential work that could not be carried out at home and once a day for exercise. People considered at increased risk of serious complications following infection were advised to “self-isolate” (i.e. limit outside contact and not leave their homes). One such at risk population is Rheumatoid Arthritis (RA), which is an autoimmune disease characterised by inflammation, pain, fatigue and poor mental health, and commonly managed by immunosuppressive therapies [2, 3]. Whilst restrictions were deemed necessary to contain the spread of the virus, they can negatively impact mental health and psychological wellbeing [4], as well as behaviours which can support mental health and wellbeing such as physical activity (PA) and sedentary behaviour [5].

High levels of anxiety, depression and stress have been reported in the general population during COVID-19 restrictions [6–9]. In people living with rheumatic diseases, a study conducted in the United States has reported difficulty managing negative emotions, perceived increased risk of being infected and reduced access to healthcare and medications during this pandemic [10]. Such COVID-19-related concerns have been associated with poor mental health and compromised psychological wellbeing in the general population [8, 9]. As the mental health impact of COVID-19 is even greater in those self-isolating [4, 11], people living with RA, a population already at risk of compromised mental health [12], may be at even greater risk of adverse psychological impact [5]. Indeed, negative consequences of COVID-19 on mental health has been reported in 73% participants with rheumatic diseases in one study [13]. Therefore, identifying factors that could positively impact mental health during the COVID-19 pandemic is critical for establishing effective management to attenuate the negative impact of this pandemic on wellbeing.

In the general population, PA is positively associated with indicators of mental health and psychological wellbeing and reductions in PA are associated with negative mental health during COVID-19 [11]. In RA, PA is related to reduced anxiety, depression, fatigue, pain and increased vitality [3, 14–17]. However, the COVID-19 lockdown has restricted opportunities for PA and not surprisingly, lower PA and increased screen time have been reported [11, 18]. This may pose a significant risk for the mental health and psychological wellbeing of those, who are already at risk of low levels of PA and high levels of sedentary behaviour (i.e. waking activities involving sitting/lying and energy expenditure ≤ 1.5 metabolic equivalents), such as people with RA [19]. Indeed, during COVID-19, people with rheumatic diseases reported challenges to being active [10] and reductions in PA and increased sedentary time (ST) have been reported in RA [20], which could further worsen mental health and wellbeing [21].

Emerging evidence during COVID-19 emphasises the benefits of PA for mental health in the general population [8, 22, 23]. However, associations between PA, mental health and psychological wellbeing during COVID-19 have not been investigated in RA. The aims of this study were to (1) explore the associations between PA and ST with indicators of mental health and wellbeing in RA during COVID-19 and (2) examine the impact of self-isolation on these associations.

## Methods

### Participants

People with RA were recruited through social media of the National Rheumatoid Arthritis Society (NRAS) in the UK. Inclusion criteria were a self-reported clinical diagnosis of RA and aged ≥ 18 years. Ethical approval was obtained from the University of Birmingham ethics committee (ERN_20-0475). Participants were given an information sheet, provided informed consent and completed the online questionnaire between April 8 and April 30 2020, when the most stringent lockdown restrictions applied in the UK.

### Patient and public involvement

This online questionnaire was developed in collaboration with NRAS.

### Measures

#### Physical activity and sedentary behaviour

PA was assessed using the National Institutes of Health-American Association of Retired Persons (NIH-AARP) Diet and Health Study questionnaire to record participation in different types of PA [24]. Participants were asked to indicate how much time they had spent during the last 7 days in 3 PA types: (1) light PA (LPA) (e.g. cooking, laundry), (2) walking and (3) exercise (e.g. tennis, cycling). Items were scored using a categorical scale with response options from “none” to “ ≥ 10 h”. Sedentary behaviour was assessed using the International Physical Activity Questionnaire-Short Form (IPAQ-SF), which comprises 2 questions. Participants are asked “during the last 7 days, how much time did you spend sitting on a”…. (1) weekday and (2) weekend day, to calculate overall weekly ST. Both questionnaires are reliable and valid measures of PA and ST in older adults [24, 25].

#### Pain

Pain experienced during the past week was assessed with the McGill Pain Questionnaire (MPQ), comprising 15 pain descriptors (e.g. “throbbing” and “tender”) on a scale from 0 (none) to 3 (severe) [26]. Participants were also asked to rate last week’s pain on a visual analogue scale (VAS), from 0 (no pain) to 10 (worst imaginable pain) [27]. The MPQ demonstrated good internal reliability in this study (Cronbach *α* = .92) and the MPQ and VAS have been validated in RA [28].

#### Fatigue

Fatigue during the past week was assessed using the Multidimensional Fatigue Inventory (MFI) reflecting physical, mental and general fatigue (4 items each), rated on a scale from 1 (no, that’s not true) to 5 (yes, that’s true) [29]. MFI is a validated fatigue measure used in RA, with good internal reliability in this study (physical:* α* = .78, mental:* α* = .86, general:* α* = .73) [30].

#### Anxious and depressive symptoms

Anxious (7 items) and depressive (7 items) symptoms during the past week were measured using the Hospital Anxiety and Depression Scale (HADS) [31]. Participants were asked to indicate their agreement (on a scale from 0 to 3) with each statement (e.g. “I feel tense or ‘wound-up’”). The HADS has previously shown good validity [32] and good internal reliability (anxious symptoms: *α* = .87, depressive symptoms: *α* = .83) in this study.

#### Subjective vitality

Vitality, a measure of positive wellbeing, experienced during the past week was measured using the Subjective Vitality Scale (SVS) [33]. Participants were asked to rate 6 statements (e.g. “I’ve been feeling energised”) on a scale from 1 (not at all true) to 7 (very true). The SVS demonstrates good reliability in this study (*α* = .88) and has been validated in RA [17].

#### Functional disability

Participants’ functional disability was determined using the Stanford Health Assessment Questionnaire disability index (HAQ-DI), comprising 8 subscales each reflecting an activity of daily living (i.e. dressing, rising, eating, walking, hygiene, grip, reach and activities) [34]. Participants were asked to rate their ability to perform specific activities (e.g. “open car doors”) on a scale from “without any difficulty” to “unable to do” and indicate if they used aids/devices for each activity. An overall disability index score is calculated as an average of the 8 subscales. A higher score represents higher functional disability.

#### General COVID-19 concern

Concerns regarding COVID-19 were measured as the extent to which participants were apprehensive about (1) testing positive for COVID-19, (2) a family member testing positive for COVID-19 and (3) not being able to receive arthritis-related medical care. Each item was scored on a scale from 1 (not concerned at all) to 5 (very concerned) and the average was calculated.

#### COVID-19 living

COVID-19 Living reflected living circumstances during COVID-19, which included “self-isolating at home” (i.e. not leaving the house due to medical recommendation (i.e. shielding) or personal concern) and “not self-isolating” (i.e. leaving the house for basic necessities, exercise and/or work).

### Data reduction and statistical analysis

The data were analysed using IBM SPSS Version 26 and checked for normality using the Kolmogorov–Smirnov test. Independent variables were PA types (LPA, walking and exercise) and ST. Dependent variables were indicators of mental health and psychological wellbeing (i.e. pain (MPQ and VAS), physical, mental and general fatigue, anxious symptoms, depressive symptoms and vitality). Covariates were age, gender, functional disability, living alone/with others, education and general COVID-19 concern, all with known associations with dependent variables in RA. The moderator variable was COVID-19 Living.

408 participants provided complete data for PA and ST. Participants were excluded due to implausible ST (> 18hours/day [35], *n* = 26), missing covariate (*n* = 8) or moderator data (*n* = 6). For the dependent variables, the missing data were imputed using the expectation maximisation method where participants were missing one item of a questionnaire (MPQ: *n* = 24; MFI: *n* = 15; HADS: *n* = 1; SVS: *n* = 1). Participants with > 1 missing value per questionnaire were excluded (*n* = 23). The final sample size for all statistical analysis was 345 participants.

Differences between those self-isolating vs not self-isolating were assessed with Mann–Whitney or *χ*^2^ tests, as appropriate. To address the primary aim, hierarchical linear regression analyses were conducted to examine associations between PA and ST with indicators of mental health and psychological wellbeing, while adjusting for potential covariates. In these hierarchical regression analyses, we explored the following sequential models:

Regression Model 1 examined the associations between the covariates (age, gender, functional disability, living alone/with others, education and general COVID-19 concern) with the indicators of mental health and psychological wellbeing as dependent variables (pain, physical, mental and general fatigue, anxious symptoms, depressive symptoms and vitality). Separate regression analyses were conducted for each indicator of mental health and wellbeing. For each regression analysis, the *F* value and *p* value are reported to reflect statistical significance, the *R*^2^ value is presented to reflect the variance in the indicator of mental health and psychological wellbeing explained by all covariates combined and standardised beta values (*β* values) are presented to reflect the direction and strength of the association between each covariate and indicator of mental health and psychological wellbeing.

Regression Model 2 explored the associations between LPA, walking, exercise or ST with each indicator of mental health and psychological wellbeing indicator, while adjusting for the covariates included in Model 1. In other words, Regression Model 2 expanded the analyses conducted in Model 1 (with only covariates as the predictors of mental health and psychological wellbeing) to include LPA, walking, exercise or ST (independent variable) as a predictor of the indicators of mental health and psychological wellbeing (dependent variable). Separate analyses were conducted for each combination of independent and dependent variable, with the covariates included in all regression models. For each regression analysis, Δ*R*^2^ was calculated to reflect the additional variance in the dependent variable explained by including the independent variable to the model with covariates only. *F* and *p* values are reported to reflect statistical significance of adding the independent variable to the model and *β* values reflect the direction and strength of the association between each independent (LPA, walking, exercise or ST) and dependent variable (indicator of mental health and psychological wellbeing).

Regression Model 3 explored whether the associations between the activity-related independent variables (i.e. LPA, walking or exercise) with the indicators of mental health and psychological wellbeing were independent of ST, and vice versa, whether the associations between ST and indicators of mental health and psychological wellbeing were independent of the levels of activity (LPA, walking and exercise). More specifically, where regression Model 2 revealed significant associations between LPA, walking or exercise with a specific dependent variable, Model 3 included both the significant PA type as well as ST as predictors for that dependent variable, while also adjusting for covariates. For each regression analysis, Δ*R*^2^ was calculated to reflect the additional variance explained in the dependent variable as compared to Model 2. *F* and *p* values are reported to reflect statistical significance of adding the independent variable to the model and *β* values reflected the direction and strength of the association between each independent (LPA, walking, exercise or ST) and dependent variable (indicator of mental health and psychological wellbeing).

Finally, to explore the impact of COVID-19 living situation (i.e.  self-isolating at home vs not self-isolating) on all associations between independent (LPA, walking, exercise, ST) and dependent variables (indicators of mental health and wellbeing), moderation analyses were conducted using the PROCESS model [36]. In all moderation analyses, age, gender, living situation, education, general COVID-19 concern and functional disability were included as covariates.

As the majority of variables were not normally distributed, bootstrapping was employed in regression analyses. Bootstrapping is a nonparametric re-sampling procedure that does not impose the assumption of normal distribution on the data [37]. Significance was interpreted based on the bootstrap-generated 95% bias-corrected confidence intervals (CIs) (5000 samples). CIs also provide more information than *p*-values, showing the possible variability of effect size and therefore are more appropriate for determining significance of bootstrapped data [37, 38] and standardised beta coefficients (β) were used to interpret the strength of associations.

## Results

Participant characteristics are reported in Table 1. The sample predominantly comprised white females, with moderate functional disability. Mann − Whitney and Chi-square tests revealed a longer disease duration, lower levels of LPA and walking, more pain, physical fatigue, functional disability and COVID-19 concern in self-isolating participants (all *p*’s < .05, see Table 1).

**Table 1:** Descriptive statistics with *p* values for total sample and sample stratified by COVID-19 Living status

	Self-isolating at home (*n* = 230)	Not self-isolating (*n* = 115)	All participants (*n* = 345)	*p* value
Demographic Information				
Age (years)	51.53 ± 11.82	51.37 ± 11.58	51.48 ± 11.73	.805
Gender (*n* = female (%))	214 (93.0)	107 (93.0)	321 (93.0)	1.000
Ethnicity (*n* = white (%))	224 (97.4)	110 (96.5)	334 (96.8)	.640
RA duration (years from diagnosis)	11.51 ± 10.51	8.52 ± 8.08*	10.52 ± 9.87	.012
Independent variables				
LPA (minutes/week)	150 ± 420	300 ± 420*	300 ± 420	.033
Walking (minutes/week)	60 ± 150	240 ± 420*	90 ± 300	< .001
Exercise (minutes/week)	0 ± 30	0 ± 30	0 ± 30	.820
Sedentary time (minutes/week)	3360 ± 1823	3360 ± 1980	3360 ± 1680	.088
Outcomes				
Pain (MPQ)	15.34 ± 9.30	13.12 ± 9.02*	14.60 ± 9.26	.024
Pain (VAS rating)	4.57 ± 2.62	4.10 ± 2.58	4.41 ± 2.62	.095
Physical fatigue	15.25 ± 4.06	14.06 ± 4.15*	14.85 ± 4.12	.007
Mental fatigue	12.04 ± 5.00	11.94 ± 5.01	12.01 ± 5.00	.860
General fatigue	15.88 ± 3.63	15.31 ± 4.21	15.69 ± 3.83	.383
Anxious symptoms	9.39 ± 4.58	8.34 ± 4.51	9.04 ± 4.58	.055
Depressive symptoms	7.39 ± 3.94	6.86 ± 4.04	7.21 ± 3.98	.146
Subjective Vitality	2.56 ± 1.20	2.74 ± 1.32	2.62 ± 1.24	.266
Covariates				
Functional disability (HAQ-DI)	1.33 ± 0.77	0.99 ± 0.69*	1.22 ± 0.76	< .001
Living situation (*n* = living alone (%))	43 (18.7)	17 (14.8)	60 (17.4)	.366
Education (*n* = higher education (%))	119 (51.7)	61 (53.0)	180 (52.2)	.819
General COVID-19 Concern	3.91 ± 0.90	3.62 ± 0.90*	3.81 ± 0.91	.003

Values are reported as means ± SD, except for PA and ST variables which show medians ± IQR

Living situation was characterised as living with others (i.e. partner, family) or living alone. Education was characterised as higher education (university degree, doctorate) or secondary education (GCSE/O-level, A-level/GCE)

*RA *rheumatoid arthritis, *LPA* light physical activity, *MPQ* McGill Pain Questionnaire, *VAS* Visual Analogue Scale, *HAQ-DI* Health Assessment Questionnaire-Disability Index, *SD* standard deviation, *IQR* interquartile rage

^*^Significantly different from self-isolating at home with *p* < .05. Differences were examined using Mann–Whitney *U* and *χ*^2^ tests, as appropriate

### Regression analyses

Model 1: The associations between covariates with each mental health and psychological wellbeing indicator are summarised in Table 2. As is evident from Table 2, all regression models were statistically significant. Examination of the *β* values of the covariates showed that functional disability was most strongly and consistently associated with all indicators of mental health and psychological wellbeing, with higher functional disability related to more pain, fatigue and depressive and anxious symptoms and lower vitality.

**Table 2:** Model 1 Regression Analyses for all covariates with each indicator of mental health and psychological wellbeing (dependent variable)

	Pain (MPQ)	Pain (VAS rating)	Physical Fatigue	Mental Fatigue	General Fatigue	Anxious Symptoms	Depressive Symptoms	Vitality
	*R*^2^ = .383	*R*^2^ = .330	*R*^2^ = .348	*R*^2^ = .140	*R*^2^ = .260	*R*^2^ = .207	*R*^2^ = .219	*R*^2^ = .176
	*F* = 34.91	*F* = 27.73	*F* = 30.09	*F* = 9.15	*F* = 19.78	*F* = 14.67	*F* = 15.77	*F* = 12.05
	*p* < .001	*p* < .001	*p* < .001	*p* < .001	*p* < .001	*p* < .001	*p* < .001	*p* < .001
	*β*	*β*	*β*	*β*	*β*	*β*	*β*	*β*
Age	− .11*	− .08	− .11*	− .27*	− .21*	− .25*	− .16*	.18*
Gender	− .01	− .02	.05	− .06	− .03	− .01	.01	− .03
Education	− .03	.01	.05	− .04	.03	− .10*	− .04	.06
Living Situation	.02	.04	.01	− .10*	.01	.02	− .09	.07
Concern	.15*	.08	.09	.10	.04	.32*	.15*	− .08
Functional Disability	.55*	.55*	.55*	.23*	.46*	.14*	.38*	− .34*

Model 1: Regressions included all covariates (age, gender, education, living situation, general COVID-19 concern and functional disability) as predictors for each indicator of mental health and wellbeing in separate analyses

*R*^2^ represents the variance explained in the dependent variable by all covariates together. Statistical information about each model is presented by the *F*-value and the *p*-value, with *β* representing the standardised beta coefficient of each covariate

*Concern* general COVID-19 concern, *MPQ* McGill Pain Questionnaire, *VAS* Visual Analogue Scale, *CI* confidence interval

^*^Significant associations between covariates with indicators of mental health and wellbeing derived using bootstrapped 95% CI

Model 2 expanded on Model 1 with the covariates only, by adding the independent variables (LPA, walking, exercise, ST) as predictors of the indicators of mental health and psychological wellbeing in separate analyses. Table 3 presents the summary findings of Model 2 regression analyses, by focusing on the additional amount of variance explained by each independent variable and the associated β-coefficient. More detailed information on these models, including all covariates, unstandardised beta values (B) and the 95% CI (used to assess significance of the model), is reported in Supplementary Tables S1–S4. LPA was significantly negatively associated with mental fatigue and depressive symptoms and positively with vitality. Walking was negatively related to physical fatigue and depressive symptoms and positively with vitality. Exercise was negatively associated with physical and general fatigue and depressive symptoms. ST was positively linked to physical fatigue. No other significant associations were detected. In all these analyses, the β-coefficients for the covariates remained broadly similar to those reported in Table 2. More detailed information about the full regression models is presented in Supplementary Tables S1–S4.

**Table 3:** **Summary** Model 2 Regression Analyses for LPA, Walking, Exercise and Sedentary Time with dependent variables adjusting for covariates

	Pain (MPQ)		Pain (VAS rating)		Physical Fatigue		Mental Fatigue		General Fatigue		Anxious Symptoms		Depressive Symptoms		Vitality	
	β	Δ*R*^2^	*β*	Δ*R*^2^	*β*	Δ*R*^2^	*β*	Δ*R*^2^	*β*	Δ*R*^2^	*β*	Δ*R*^2^	*β*	Δ*R*^2^	*β*	Δ*R*^2^
LPA	− .02	.000	− .06	.003	− .08	.006	− .11*	.011†	− .07	.005	− -.04	.002	-.14*	.018†	.13*	.016†
Walking	.04	.002	.06	.003	− .11*	.010†	− .01	.000	− .06	.003	.02	.000	-.12*	.012†	.15*	.020†
Exercise	.04	.001	.01	.000	− .19*	.033†	− .04	.001	− .12*	.013†	− .04	.001	− .09*	.008	.07	.005
Sedentary Time	− .08	.006	− .02	.000	.19*	.032†	.04	.001	.07	.005	− .04	.001	.08	.006	− .09	.008

Model 2: expanded Model 1 (covariates age, gender, education, living situation, general COVID-19 concern and functional disability only) by adding LPA, walking, exercise and ST as individual predictors for each dependent variable.

*β* represents the standardised beta coefficient and Δ*R*^2^ represents the proportion of the variance that is explained by the addition of the predictor independent variable to the model relative to Model 1.  β coefficients for covariates were broadly similar as those reported in Table 1. Therefore, to improve readability, *β*'s are only reported for the associations between the independent variables (LPA, walking, exercise, ST) and the dependent variables. Full information of all Model 2 regressions, including the 95% CI intervals to determine significance, *F*-value with associated *p-*values and the *β* for each covariate are reported in Supplementary Tables S1-S4

*LPA* light physical activity, *Concern* general COVID-19 concern, *MPQ* McGill Pain Questionnaire, *VAS* Visual Analogue Scale, *CI* confidence interval

^*^significant *β* (standardised beta) coefficients derived using bootstrapped 95% CI (see Supplementary Tables S1–S4); †*p* < .05 for Δ*R*^2^ values determining the significance of the overall model

Model 3 explored whether the associations reported between the activity-related independent variables with the dependent variables were independent of levels of ST and vice versa. When adding ST as an additional predictor, all significant associations between activity-related independent variables with the indicators of mental health and psychological wellbeing observed in Model 2 remained significant, with the exception of the association between walking with physical fatigue, which no longer remained significant when ST was added to the model (*β* = − .08, *B* = − 0.001, 95% CI = − 3.1∙10^–3^, 1.5∙10^–4^). In both walking and exercise models, ST was significantly associated with physical fatigue (walking model: *β* = .18, *B* < 0.001, 95% CI = 2.8∙10^–4^, 8.3∙10^–4^; exercise model: *β* = .18, *B* < 0.001, 95% CI = 2.9∙10^–4^, 8.2∙10^–4^).

### Moderation analyses

Moderation analyses revealed that COVID-19 living situation only moderated the associations between LPA with mental fatigue and vitality and walking with physical fatigue. More LPA was significantly associated with lower mental fatigue and better vitality in those who were not self-isolating (mental fatigue model: *β* = − .26, 95% CI = − .42, − .09; vitality model: *β* = .31, 95% CI = .15, .47), but not in those who were self-isolating (mental fatigue model: *β* = − .04, 95% CI = − .16, .10; vitality model: *β* = .03, 95% CI = − .09, .16). Walking was associated with lower physical fatigue in people who were self-isolating (*β* = − .22, 95% CI = − .35, − .08), but not in those not self-isolating (*β* = − .02, 95% CI = − .15, .12). For more detailed information of moderation analysis results, please see Supplementary Table S5.

## Discussion

This is the first study to show associations between activity behaviours with indicators of mental health and psychological wellbeing in people with RA during COVID-19. LPA and walking were associated with lower physical and mental fatigue and depressive symptoms and higher vitality. Exercise was related to lower physical and general fatigue and fewer depressive symptoms and ST was related to higher physical fatigue. In addition, COVID-19 living situation moderated some associations between LPA and walking with physical and mental fatigue and vitality.

The finding that LPA and walking were associated with higher vitality is in line with epidemiological research demonstrating a relationship between LPA with wellbeing in older adults [39]. Our results point to the importance of LPA and walking for wellbeing in RA during COVID-19. From a behaviour change perspective, encouraging non-exercise LPA (e.g. household chores) and walking may be perceived as more feasible and accessible for RA, a population experiencing significant disease-related barriers to PA [40].

PA was associated with fewer depressive symptoms, aligned with the previous arthritis research [2, 15]. Associations were of a similar, although opposite, magnitude to associations between COVID-19 concerns and depressive symptoms. Fear or concern about the virus has been related to depression [4], and our findings suggest that PA counteracts this negative impact on depressive symptoms in RA, in line with findings in college students and older adults [22, 41]. Importantly, associations between PA with depressive symptoms and vitality were independent of functional disability in this study and others [2]. Thus, the activity at any intensity should be promoted in all people with RA to improve mental health and wellbeing, regardless of functional disability.

PA was associated with lower and ST with higher physical and mental fatigue, in line with interventions promoting PA and reducing ST improving fatigue in RA [3, 42, 43]. Our findings emphasise the importance of the multidimensional aspects of fatigue in RA. Specifically, LPA was negatively associated with mental fatigue, whereas walking, exercise and ST were related to physical fatigue. Our results suggest that different PA types could be related to different aspects of fatigue in people living with RA. Aligned with present findings, exercise interventions have been reported to be particularly effective for physical fatigue in RA [30].

PA can lead to improved mental health and wellbeing during lockdown situations through several pathways. For example, PA can distract from negative thoughts and worries [44], being active can have an immediate positive effect on mood [44], outdoor environment can induce mental stimulation [23] and PA can provide structure when daily routine is disrupted due to lockdown, with the resulting sense of control improving wellbeing [45]. Thus, recommendations promoting PA may offer an avenue to support clinical populations to cope with the impact of COVID-19 on their mental health [41, 46]. Given that maintaining PA during COVID-19 is associated with better mental health in older adults [41], the present results imply that increasing PA may positively impact mental health and wellbeing in RA during COVID-19. Given the duration of the pandemic and recent return of restrictions, longitudinal studies during COVID-19 are needed to understanding how changes in PA contribute to better mental health and wellbeing in RA. This can inform guidance on management of wellbeing during these difficult times, not just in RA, but also other clinical populations.

PA and ST were not associated with anxious symptoms in this study. There are mixed findings related to anxiety and PA during COVID-19 [22, 23, 47]. The impact of PA on anxiety during COVID-19 may be influenced by prior activity levels. Indeed, research suggests inactive people who increased PA during COVID-19 reported lower anxiety compared to those who became less active, but these associations were not seen in people who were classed as active prior to COVID-19 [23]. Thus, perhaps instead of looking at absolute values, it might be more important to examine changes in PA during a pandemic in relation to anxiety.

People with RA in this study did not report associations between PA and ST with pain, which is in contrast to previous observational studies of RA [48]. This may be due to the possible bi-directional association between PA with mental health and wellbeing [49] and also the association between pain and functional disability [50], which may have affected levels of PA observed depending on COVID-19 living status. Specifically, correlational analysis revealed walking was negatively related to pain (data not reported), but this association was no longer significant when adjusting for functional disability. Given our findings suggested that people with RA who were self-isolating had higher levels of pain and functional disability and lower levels of LPA and walking, compared to those leaving the house, it could be assumed that individuals with the least pain and disability were more likely to leave the house for PA. This lower variability in pain among those who were accruing some form of PA (through leaving the house), could mean associations between PA and pain are less likely to be observed.

The lower levels of PA observed among people with RA who were self-isolating, is in agreement with previous studies [11]. Moderation analysis showed LPA was only related to mental fatigue and vitality in those not self-isolating, whereas walking was only associated with physical fatigue in those self-isolating. As those self-isolating did significantly less walking than those not self-isolating, this could suggest that walking specifically, should be encouraged among individuals self-isolating to reduce physical fatigue. However, as few significant moderation effects of COVID-19 living were observed, these findings should be interpreted with care.

Except for physical fatigue, ST was not associated with indicators of mental health or wellbeing, contrasting previous COVID-19 research in the general population [11]. Our measure of ST reflected total ST and did not differentiate between different sedentary behaviours; e.g. sitting while being intellectually stimulated, e.g. during work, is suggested to have a less negative impact health and wellbeing compared to sitting watching TV or using electronic devices [51]. Consequently, future studies are required to understand the specific role of different sedentary behaviours for mental health and wellbeing in RA, both during and beyond the pandemic.

The current study included a large sample of people with RA during stringent lockdown conditions. Therefore, it was only possible to collect self-report data for all outcomes, which should be acknowledged as a limitation. In addition, the associations reported are cross-sectional, so causality cannot be inferred. Therefore, exploring these associations over time during COVID-19 will help to better understand the implications of this pandemic on the link between activity behaviours and mental health and wellbeing and how PA can support mental health and wellbeing throughout the pandemic.

In summary, PA, specifically LPA and walking, was positively associated with mental health and psychological wellbeing in RA during COVID-19. These findings support recommendations from different governments to encourage PA during lockdown restrictions, to attenuate the negative impact of a pandemic on mental health and wellbeing. Given the known barriers for PA in RA [40] and the reported additional barriers experienced during COVID-19 [10], these findings emphasise the importance of appropriate support and recommendations for PA in people with RA and potentially other clinical populations, particularly in those self-isolating, during a pandemic to maintain mental health and psychological wellbeing.

## Electronic supplementary material

Below is the link to the electronic supplementary material.

Supplementary file1 (DOCX 61 KB)
