# Supplementary material for: Different types of physical activity are positively associated with indicators of mental health and psychological wellbeing in rheumatoid arthritis during COVID-19
Source: Rheumatol Int. 2020 Nov 30;41(2):335–44. doi: 10.1007/s00296-020-04751-w (PMC7703721; doi:10.1007/s00296-020-04751-w)
Supplement: Supplementary file 1 — Supplementary file1 (DOCX 61 KB) [file 296_2020_4751_MOESM1_ESM.docx]

**Title:** Different Types of Physical Activity are Positively Associated with Indicators of Mental Health and Psychological Wellbeing in Rheumatoid Arthritis during COVID-19

**Journal Name:** Rheumatology International

**Authors:** Sophia M. Brady^1,2,3^, Sally A.M. Fenton^1,2,3^, George S. Metsios^2,4^, Ailsa Bosworth^5^, Joan L. Duda^1,3^, George D. Kitas^1,2^, Jet J.C.S. Veldhuijzen van Zanten^1,2,3^

**Corresponding Author**

Jet J.C.S. Veldhuijzen van Zanten – veldhujj@bham.ac.uk

School of Sport, Exercise and Rehabilitation Sciences

University of Birmingham

Birmingham

B15 2TT

United Kingdom

**Co-Authors:**

Sophia M. Brady (SB) – [sxb1075@student.bham.ac.uk](mailto:sxb1075@student.bham.ac.uk)

Sally A.M. Fenton (SF) – s.a.m.fenton@bham.ac.uk

George S. Metsios (GM) – g.metsios@wlv.ac.uk

Ailsa Bosworth (AB) – [ailsa@nras.org.uk](mailto:ailsa@nras.org.uk)

Joan L. Duda (JD) – j.l.duda@bham.ac.uk

George D. Kitas (GK) – [George.kitas@nhs.net](mailto:George.kitas@nhs.net)

Jet J.C.S. Veldhuijzen van Zanten (JVvZ) – veldhujj@bham.ac.uk

^1^ School of Sport, Exercise and Rehabilitation Sciences, University of Birmingham, Birmingham,

United Kingdom

^2^ Rheumatology Department, Dudley Group NHS Foundation Trust, Dudley, United Kingdom

^3^ Medical Research Council- Versus Arthritis Centre for Musculoskeletal Ageing, University of Birmingham, Birmingham, United Kingdom

^4^ Faculty of Education, Health and Wellbeing, University of Wolverhampton, United Kingdom

^5^ National Rheumatoid Arthritis Society, United Kingdom

**ACKNOWLEDGEMENTS**

The authors would like to thank the National Rheumatoid Arthritis Society (NRAS) for their support in the development of this study and the recruitment for this study. In addition, the authors would like to thank all participants for their time to complete to questionnaire pack.

**Supplementary Table S1**: Model 2 Regression Analyses for Light non-exercise PA (min/week) with indicators of mental health and psychological wellbeing

|  | **Pain (MPQ)** | | | **Pain (VAS rating)** | | | **Physical Fatigue** | | | **Mental Fatigue** | | | **General Fatigue** | | | **Anxious Symptoms** | | | **Depressive Symptoms** | | | **Vitality** | | |
| --- | --- | --- | --- | --- | --- | --- | --- | --- | --- | --- | --- | --- | --- | --- | --- | --- | --- | --- | --- | --- | --- | --- | --- | --- |
|  | R²= .383 | | | R²= .333 | | | R²= .354 | | | R²= .151 | | | R²= .264 | | | R²= .208 | | | R²= .237 | | | R²= .192 | | |
|  | ΔR^2^= .000 | | | ΔR^2^= .003 | | | ΔR^2^= .006 | | | ΔR^2^= .011 | | | ΔR^2^= .005 | | | ΔR^2^= .002 | | | ΔR^2^= .018 | | | ΔR^2^= .016 | | |
|  | F= 0.23, p=.63 | | | F= 1.66, p=.20 | | | F= 2.88, p=.09 | | | F= 4.40, p=.04 | | | F= 2.14, p=.15 | | | F= 0.77, p=.38 | | | F= 8.03, p=.01 | | | F= 6.69, p=.01 | | |
|  | **β** | **B** | **95% CI** | **β** | **B** | **95% CI** | **β** | **B** | **95% CI** | **β** | **B** | **95% CI** | **β** | **B** | **95% CI** | **β** | **B** | **95% CI** | **β** | **B** | **95% CI** | **β** | **B** | **95% CI** |
| Age | -.11* | -0.08* | -0.15, -0.01 | -.08 | -0.02 | -0.04, 0.00 | -.10* | -0.03* | -0.07, 0.00 | -.25* | -0.11* | -0.15, -0.07 | -.21* | -0.07* | -0.10, -0.03 | -.24* | -0.09* | -0.13, -0.05 | -.14* | -0.05* | -0.08, -0.02 | .16* | 0.02* | 0.01, 0.03 |
| Gender | -.01 | -0.26 | -3.29, 2.69 | -.02 | -0.19 | -1.19, 0.75 | .06 | 0.97 | -0.50, 2.42 | -.05 | -1.04 | -2.85, 0.67 | -.03 | -0.42 | -1.73, 0.95 | -.01 | -0.15 | -1.65, 1.35 | .03 | 0.39 | -1.17, 1.89 | -.05 | -0.23 | -0.65, 0.15 |
| Education | -.03 | -0.58 | -2.19, 1.05 | .01 | 0.08 | -0.40, 0.54 | .06 | 0.51 | -0.23, 1.26 | -.03 | -0.32 | -1.33, 0.71 | .04 | 0.29 | -0.43, 1.02 | -.09 | -0.86 | -1.77, 0.04 | -.03 | -0.23 | -0.99, 0.52 | .05 | 0.12 | -0.13, 0.37 |
| Living Situation | .03 | 0.60 | -1.59, 2.73 | .05 | 0.37 | -0.23, 0.94 | .03 | 0.30 | -0.56, 1.19 | -.08 | -1.12 | -2.46, 0.19 | .02 | 0.23 | -0.65, 1.14 | .03 | 0.32 | -0.84, 1.42 | -.07 | -0.73 | -1.71, 0.25 | .04 | 0.14 | -0.19, 0.45 |
| Concern | .15* | 1.57* | 0.74, 2.39 | .09 | 0.25 | -0.02, 0.50 | .09 | 0.40 | -0.02, 0.80 | .10 | 0.54 | -0.04, 1.09 | .04 | 0.16 | -0.27, 0.57 | .32* | 1.60* | 1.11, 2.07 | .15* | 0.66* | 0.20, 1.07 | -.08 | -0.11 | -0.25, 0.03 |
| Functional Disability | .55* | 6.74* | 5.67, 7.81 | .54* | 1.86* | 1.56, 2.15 | .54* | 2.95* | 2.46, 3.44 | .22* | 1.43* | 0.77, 2.09 | .46* | 2.31* | 1.82, 2.82 | .13* | 0.79* | 0.18, 1.40 | .36* | 1.90* | 1.34, 2.45 | -.33* | -0.54* | -0.71,  -0.38 |
| **LPA** | -.02 | -0.00 | -5.2∙10⁻³, 3.2∙10⁻³ | -.06 | -0.00 | -1.9∙10⁻³, 3.8∙10⁻⁴ | -.08 | -0.00 | -3.4∙10⁻³, 2.8∙10⁻⁴ | -.11* | -0.00* | -5.2∙10⁻³,  -1.5∙10⁻⁴ | -.07 | -0.00 | -3.2∙10⁻³, 4.1∙10⁻⁴ | -.04 | -0.00 | -3.3∙10⁻³, 1.4∙10⁻³ | -.14* | -0.00* | -4.8∙10⁻³,  -6.4∙10⁻⁴ | .13* | 0.00* | 1.9∙10⁻⁴, 1.4∙10⁻³ |

Note: Significant associations (=*) between LPA with indicators of mental health and well-being were interpreted using bootstrapped 95% CI. Bootstrapping was used to compute 95% CI which produces an unstandardised B-coefficient and corresponding unstandardised 95% CI. The standardised beta-value (β) is reported (and in the main analyses) to allow to facilitate the interpretation regarding the strength and direction of all associations reported.

Model 2: expanded Model 1 (covariates age, gender, education, living situation, general COVID-19 concern and functional disability only) by adding LPA as a predictor of the separate indicators of mental health and psychological wellbeing.

R² represents the variance explained on the dependent variable (pain, fatigue, anxious and depressive symptoms and vitality) by LPA (independent variable) and all covariates together. ΔR^2^ indicates proportion of the variance that is explained by the addition of the LPA to the Model 1 (covariates only). The F value and p statistic represent the f-statistic related to the ΔR², indicating the significance of the model when adding the additional predictor (i.e., significance of the ΔR²). β represents the standardised beta coefficient.

B= unstandardised beta coefficient, 95% CI= 95% Confidence Interval [lower limit, upper limit], LPA= light physical activity, Concern= General COVID-19 Concern, MPQ= McGill Pain Questionnaire, VAS= Visual Analogue Scale.

The associations between covariates and indicators of mental health and well-being were unchanged from Model 1 (Δβ<0.05). There were minor significance changes for some associations that were borderline significant in Model 1: between age and physical fatigue, education and anxious symptoms and living situation and mental fatigue.

**Supplementary Table S2**: Model 2 Regression Analyses for Walking PA (min/week) with dependent variables

|  | **Pain (MPQ)** | | | **Pain (VAS rating)** | | | **Physical Fatigue** | | | **Mental Fatigue** | | | **General Fatigue** | | | **Anxious Symptoms** | | | **Depressive Symptoms** | | | **Vitality** | | |
| --- | --- | --- | --- | --- | --- | --- | --- | --- | --- | --- | --- | --- | --- | --- | --- | --- | --- | --- | --- | --- | --- | --- | --- | --- |
|  | R²= .384 | | | R²= .333 | | | R²= .358 | | | R²= .140 | | | R²= .263 | | | R²= .207 | | | R²= .231 | | | R²= .196 | | |
|  | ΔR^2^= .002 | | | ΔR^2^= .003 | | | ΔR^2^= .010 | | | ΔR^2^= .000 | | | ΔR^2^= .003 | | | ΔR^2^= .000 | | | ΔR^2^= .012 | | | ΔR^2^= .020 | | |
|  | F= 0.85, p=.36 | | | F= 1.59, p=.21 | | | F= 5.26, p=.02 | | | F= 0.02, p=.89 | | | F= 1.40, p=.24 | | | F= 0.19, p=.66 | | | F= 5.36, p=.02 | | | F= 8.33, p=.004 | | |
|  | **β** | **B** | **95% CI** | **β** | **B** | **95% CI** | **β** | **B** | **95% CI** | **β** | **B** | **95% CI** | **β** | **B** | **95% CI** | **β** | **B** | **95% CI** | **β** | **B** | **95% CI** | **β** | **B** | **95% CI** |
| Age | -.11* | -0.09* | -0.16, -0.02 | -.09 | -0.02 | -0.04, 0.00 | -.10* | -0.03* | -0.07, 0.00 | -.27* | -0.11* | -0.15, -0.07 | -.21* | -0.07* | -0.10, -0.04 | -.25* | -0.10* | -0.13, -0.06 | -.15* | -0.05* | -0.08, -0.02 | .17* | 0.02* | 0.01, 0.03 |
| Gender | -.01 | -0.29 | -3.29, 2.59 | -.02 | -0.23 | -1.19, 0.71 | .05 | 0.81 | -0.70, 2.31 | -.06 | -1.24 | -3.08, 0.50 | -.04 | -0.54 | -1.85, 0.85 | -.01 | -0.22 | -1.81, 1.29 | .01 | 0.15 | -1.48, 1.70 | -.03 | -0.15 | -0.57, 0.23 |
| Education | -.03 | -0.62 | -2.26, 1.02 | .01 | 0.05 | -0.41, 0.51 | .05 | 0.43 | -0.29, 1.17 | -.04 | -0.43 | -1.41, 0.56 | .03 | 0.23 | -0.50, 0.94 | -.10* | -0.90* | -1.77, -0.01 | -.04 | -0.36 | -1.09, 0.40 | .06 | 0.16 | -0.09, 0.41 |
| Living Situation | .02 | 0.48 | -1.58, 2.63 | .04 | 0.28 | -0.32, 0.90 | .02 | 0.19 | -0.66, 1.03 | -.10* | -1.37* | -2.65, -0.12 | .01 | 0.12 | -0.71, 1.01 | .02 | 0.22 | -0.87, 1.27 | -.09 | -0.95 | -1.94, 0.01 | .06 | 0.20 | -0.12, 0.52 |
| Concern | .15* | 1.58* | 0.80, 2.39 | .09 | 0.25 | -0.02, 0.51 | .08 | 0.37 | -0.04, 0.77 | .10 | 0.53 | -0.05, 1.10 | .03 | 0.15 | -0.28, 0.55 | .32* | 1.60* | 1.12, 2.06 | .14* | 0.62* | 0.20, 1.05 | -.07 | -0.10 | -0.23, 0.04 |
| Functional Disability | .57* | 6.90* | 5.83, 8.00 | .56* | 1.94* | 1.64, 2.23 | .52* | 2.83* | 2.31, 3.35 | .23* | 1.49* | 0.81, 2.21 | .45* | 2.26* | 1.75, 2.78 | .14* | 0.86* | 0.24, 1.48 | .34* | 1.80* | 1.28, 2.34 | -.30* | -0.49* | -0.67, -0.33 |
| **Walking** | .04 | 0.00 | -2.3∙10⁻³, 5.2∙10⁻³ | .06 | 0.00 | -3.7∙10⁻⁴, 1.7∙10⁻³ | -.11* | -0.00* | -3.7∙10⁻³,  -2.9∙10⁻⁴ | -.01 | 0.00 | -2.7∙10⁻³, 2.4∙10⁻³ | -.06 | -0.00 | -2.9∙10⁻³, 7.1∙10⁻⁴ | .02 | 0.00 | -1.6∙10⁻³, 2.6∙10⁻³ | -.12* | -0.00* | -3.8∙10⁻³,  -2.6∙10⁻⁴ | .15* | 0.00* | 2.5∙10⁻⁴, 1.4∙10⁻³ |

Note: Significant associations (=*) between Walking with indicators of mental health and well-being were interpreted using bootstrapped 95% CI. Bootstrapping was used to compute 95% CI which produces an unstandardised B-coefficient and corresponding unstandardised 95% CI. The standardised beta-value (β) is reported (and in the main analyses) to allow to facilitate the interpretation regarding the strength and direction of all associations reported.

Model 2: expanded Model 1 (covariates age, gender, education, living situation, general COVID-19 concern and functional disability only) by adding Walking as a predictor of the separate indicators of mental health and psychological wellbeing.

R² represents the variance explained on the dependent variable (pain, fatigue, anxious and depressive symptoms and vitality) by Walking (independent variable) and all covariates together. ΔR^2^ indicates proportion of the variance that is explained by the addition of the Walking to the Model 1 (covariates only). The F value and p statistic represent the f-statistic related to the ΔR², indicating the significance of the model when adding the additional predictor (i.e., significance of the ΔR²). β represents the standardised beta coefficient.

B= unstandardised beta coefficient, 95% CI= 95% Confidence Interval [lower limit, upper limit], Concern= General COVID-19 Concern, MPQ= McGill Pain Questionnaire, VAS= Visual Analogue Scale.

The associations between covariates and indicators of mental health and well-being were unchanged from Model 1 (Δβ<0.05). There were minor significance changes for some associations that were borderline significant in Model 1: between age and physical fatigue, education and anxious symptoms and living situation and mental fatigue.

**Supplementary Table S3**: Model 2 Regression Analyses for Exercise PA (min/week) with dependent variables

|  | **Pain (MPQ)** | | | **Pain (VAS rating)** | | | **Physical Fatigue** | | | **Mental Fatigue** | | | **General Fatigue** | | | **Anxious Symptoms** | | | **Depressive Symptoms** | | | **Vitality** | | |
| --- | --- | --- | --- | --- | --- | --- | --- | --- | --- | --- | --- | --- | --- | --- | --- | --- | --- | --- | --- | --- | --- | --- | --- | --- |
|  | R²= .384 | | | R²= .330 | | | R²= .381 | | | R²= .141 | | | R²= .273 | | | R²= .208 | | | R²= .227 | | | R²= .181 | | |
|  | ΔR^2^= .001 | | | ΔR^2^= .000 | | | ΔR^2^= .033 | | | ΔR^2^= .001 | | | ΔR^2^= .013 | | | ΔR^2^= .001 | | | ΔR^2^= .008, | | | ΔR^2^= .005 | | |
|  | F= 0.65, p=.42 | | | F= 0.04, p=.84 | | | F= 18.00, p<.001 | | | F= 0.48, p=.49 | | | F= 6.14, p=.01 | | | F= 0.62, p=.43 | | | F= 3.55, p=.06 | | | F= 2.11, p=.15 | | |
|  | **β** | **B** | **95% CI** | **β** | **B** | **95% CI** | **β** | **B** | **95% CI** | **β** | **B** | **95% CI** | **β** | **B** | **95% CI** | **β** | **B** | **95% CI** | **β** | **B** | **95% CI** | **β** | **B** | **95% CI** |
| Age | -.11* | -0.08* | -0.15, -0.01 | -.08 | -0.02 | -0.04, 0.00 | -.12* | -0.04* | -0.07, -0.01 | -.27* | -0.11* | -0.15, -0.07 | -.22* | -0.07* | -0.11, -0.04 | -.25* | -0.10* | -0.14, -0.06 | -.16* | -0.05* | -0.09, -0.02 | .18* | 0.02* | 0.01, 0.03 |
| Gender | -.01 | -0.27 | -3.25, 2.65 | -.02 | -0.25 | -1.16, 0.70 | .04 | 0.73 | -0.74, 2.29 | -.06 | -1.27 | -3.12, 0.49 | -.04 | -0.60 | -1.92, 0.83 | -.01 | -0.26 | -1.78, 1.26 | .01 | 0.13 | -1.52, 1.74 | -.03 | -0.15 | -0.58, 0.22 |
| Education | -.04 | -0.69 | -2.35, 0.90 | .01 | 0.04 | -0.43, 0.49 | .07 | 0.58 | -0.16, 1.30 | -.04 | -0.40 | -1.42, 0.63 | .04 | 0.31 | -0.40, 1.03 | -.10 | -0.87 | -1.76, 0.00 | -.04 | -0.28 | -1.05, 0.47 | .06 | 0.14 | -0.11, 0.39 |
| Living Situation | .02 | 0.51 | -1.54, 2.67 | .04 | 0.30 | -0.30, 0.89 | .01 | 0.15 | -0.72, 1.02 | -.10* | -1.38* | -2.64, -0.09 | .01 | 0.10 | -0.77, 1.00 | .02 | 0.23 | -0.92, 1.31 | -.09 | -0.99 | -1.96, -0.06 | .07 | 0.22 | -0.11, 0.52 |
| Concern | .15* | 1.57* | 0.78, 2.39 | .08 | 0.24 | -0.03, 0.49 | .08 | 0.38 | -0.03, 0.77 | .10 | 0.53 | -0.05, 1.10 | .04 | 0.15 | -0.28, 0.56 | .31* | 1.59* | 1.11, 2.06 | .15* | 0.64* | 0.20, 1.06 | -.08 | -0.11 | -0.24, 0.04 |
| Functional Disability | .56* | 6.85* | 5.73, 7.94 | .55* | 1.89* | 1.59. 2.19 | .51* | 2.79* | 2.30, 3.28 | .22* | 1.46* | 0.79, 2.18 | .44* | 2.23* | 1.74, 2.72 | .13* | 0.77* | 0.13, 1.39 | .36* | 1.87* | 1.34, 2.41 | -.33* | -0.54* | -0.71, -0.38 |
| **Exercise** | .04 | 0.00 | -3.6∙10⁻³, 1.0∙10⁻² | .01 | 0.00 | -1.6∙10⁻³, 2.0∙10⁻³ | -.19* | -0.01* | -9.7∙10⁻³,  -2.9∙10⁻³ | -.04 | -0.00 | -6.0∙10⁻³, 4.0∙10⁻³ | -.12* | -0.00* | -7.0∙10⁻³,  -1.8∙10⁻⁴ | -.04 | -0.00 | -5.5∙10⁻³, 2.3∙10⁻³ | -.09* | -0.00* | -5.5∙10⁻³,  -2.9∙10⁻⁴ | .07 | 0.00 | -3.1∙10⁻⁴, 1.9∙10⁻³ |

Note: Significant associations (=*) between Exercise with indicators of mental health and well-being were interpreted using bootstrapped 95% CI. Bootstrapping was used to compute 95% CI which produces an unstandardised B-coefficient and corresponding unstandardised 95% CI. The standardised beta-value (β) is reported (and in the main analyses) to allow to facilitate the interpretation regarding the strength and direction of all associations reported.

Model 2: expanded Model 1 (covariates age, gender, education, living situation, general COVID-19 concern and functional disability only) by adding Exercise as a predictor of the separate indicators of mental health and psychological wellbeing.

R² represents the variance explained on the dependent variable (pain, fatigue, anxious and depressive symptoms and vitality) by Exercise (independent variable) and all covariates together. ΔR^2^ indicates proportion of the variance that is explained by the addition of the Exercise to the Model 1 (covariates only). The F value and p statistic represent the f-statistic related to the ΔR², indicating the significance of the model when adding the additional predictor (i.e., significance of the ΔR²). β represents the standardised beta coefficient.

B= unstandardised beta coefficient, 95% CI= 95% Confidence Interval [lower limit, upper limit], Concern= General COVID-19 Concern, MPQ= McGill Pain Questionnaire, VAS= Visual Analogue Scale.

The associations between covariates and indicators of mental health and well-being were unchanged from Model 1 (Δβ<0.05). There were minor significance changes for some associations that were borderline significant in Model 1: between age and physical fatigue, education and anxious symptoms and living situation and mental fatigue.

**Supplementary Table S4**: Model 2 Regression Analyses for Sedentary Time (min/week) with dependent variables

|  | **Pain (MPQ)** | | | **Pain (VAS rating)** | | | **Physical Fatigue** | | | **Mental Fatigue** | | | **General Fatigue** | | | **Anxious Symptoms** | | | **Depressive Symptoms** | | | **Vitality** | | |
| --- | --- | --- | --- | --- | --- | --- | --- | --- | --- | --- | --- | --- | --- | --- | --- | --- | --- | --- | --- | --- | --- | --- | --- | --- |
|  | R²= .389 | | | R²= .330 | | | R²= .380 | | | R²= .141 | | | R²= .265 | | | R²= .208 | | | R²= .225 | | | R²= .184 | | |
|  | ΔR^2^= .006 | | | ΔR^2^= .000 | | | ΔR^2^= .032 | | | ΔR^2^= .001 | | | ΔR^2^= .005 | | | ΔR^2^= .001 | | | ΔR^2^= .006 | | | ΔR^2^= .008 | | |
|  | F= 3.43, p=.07 | | | F= 0.12, p=.73 | | | F= 17.42, p<.001 | | | F= 0.51, p=.48 | | | F= 2.20, p=.14 | | | F= 0.58, p=.45 | | | F= 2.74, p=.10 | | | F= 3.12, p=.08 | | |
|  | **β** | **B** | **95% CI** | **β** | **B** | **95% CI** | **β** | **B** | **95% CI** | **β** | **B** | **95% CI** | **β** | **B** | **95% CI** | **β** | **B** | **95% CI** | **β** | **B** | **95% CI** | **β** | **B** | **95% CI** |
| Age | -.13* | -0.10* | -0.17, -0.03 | -.09 | -0.02 | -0.04, 0.00 | -.07 | -0.02 | -0.06, 0.01 | -.26* | -0.11* | -0.15, -0.07 | -.20* | -0.07* | -0.10, -0.03 | -.25* | -0.10* | -0.14, -0.06 | -.14* | -0.05* | -0.08, -0.02 | .16* | 0.02* | 0.01, 0.03 |
| Gender | -.01 | -0.40 | -3.16, 2.40 | -.02 | -0.25 | -1.22, 0.70 | .06 | 0.93 | -0.57, 2.48 | -.06 | -1.22 | -3.11, 0.49 | -.03 | -0.49 | -1.80, 0.90 | -.01 | -0.24 | -1.82, 1.30 | .01 | 0.23 | -1.35, 1.79 | -.04 | -0.18 | -0.59, 0.21 |
| Education | -.03 | -0.49 | -2.07, 1.10 | .01 | 0.05 | -0.40, 0.53 | .04 | 0.30 | -0.41, 1.04 | -.05 | -0.46 | -1.50, 0.56 | .02 | 0.18 | -0.55, 0.89 | -.10 | -0.87 | -1.75, -0.01 | -.05 | -0.41 | -1.16, 0.36 | .07 | 0.18 | -0.08, 0.43 |
| Living Situation | .00 | 0.11 | -2.00, 2.12 | .04 | 0.28 | -0.35, 0.88 | .05 | 0.56 | -0.27, 1.41 | -.10 | -1.27 | -2.58, -0.04 | .02 | 0.25 | -0.65, 1.15 | .01 | 0.14 | -0.97, 1.25 | -.08 | -0.81 | -1.75, 0.13 | .05 | 0.16 | -0.17, 0.46 |
| Concern | .15* | 1.57* | 0.73, 2.36 | .08 | 0.24 | -0.02, 0.50 | .09 | 0.39 | -0.01, 0.78 | .10 | 0.53 | -0.05, 1.13 | .04 | 0.16 | -0.28, 0.57 | .32* | 1.60* | 1.10, 2.07 | .15* | 0.64* | 0.20, 1.07 | -.08 | -0.11 | -0.24, 0.03 |
| Functional Disability | .57* | 6.97* | 5.88, 8.04 | .55* | 1.89* | 1.58, 2.19 | .51* | 2.79* | 2.29, 3.27 | .22* | 1.46* | 0.76, 2.14 | .45* | 2.27* | 1.77, 2.79 | .14* | 0.87* | 0.25, 1.47 | .36* | 1.88* | 1.35, 2.42 | -.33* | -0.53* | -0.70, -0.37 |
| **Sedentary Time** | -.08 | -0.00 | -1.2∙10⁻³, 5.9∙10⁻⁵ | -.02 | -0.00 | -2.2∙10⁻⁴, 1.6∙10⁻⁴ | .19* | 0.00* | 3.3∙10⁻⁴, 8.4∙10⁻⁴ | .04 | 0.00 | -2.6∙10⁻⁴, 5.4∙10⁻⁴ | .07 | 0.00 | -4.6∙10⁻⁵, 4.8∙10⁻⁴ | -.04 | 0.00 | -5.0∙10⁻⁴, 2.2∙10⁻⁴ | .08 | 0.00 | -4.3∙10⁻⁵, 5.4∙10⁻⁴ | -.09 | -0.00 | -1.8∙10⁻⁴, 1.1∙10⁻⁵ |

Note: Significant associations (=*) between Sedentary Time with indicators of mental health and well-being were interpreted using bootstrapped 95% CI. Bootstrapping was used to compute 95% CI which produces an unstandardised B-coefficient and corresponding unstandardised 95% CI. The standardised beta-value (β) is reported (and in the main analyses) to allow to facilitate the interpretation regarding the strength and direction of all associations reported.

Model 2: expanded Model 1 (covariates age, gender, education, living situation, general COVID-19 concern and functional disability only) by adding Sedentary Time as a predictor of the separate indicators of mental health and psychological wellbeing.

R² represents the variance explained on the dependent variable (pain, fatigue, anxious and depressive symptoms and vitality) by Sedentary Time (independent variable) and all covariates together. ΔR^2^ indicates proportion of the variance that is explained by the addition of the Sedentary Time to the Model 1 (covariates only). The F value and p statistic represent the f-statistic related to the ΔR², indicating the significance of the model when adding the additional predictor (i.e., significance of the ΔR²). β represents the standardised beta coefficient.

B= unstandardised beta coefficient, 95% CI= 95% Confidence Interval [lower limit, upper limit], Concern= General COVID-19 Concern, MPQ= McGill Pain Questionnaire, VAS= Visual Analogue Scale.

The associations between covariates and indicators of mental health and well-being were unchanged from Model 1 (Δβ<0.05). There were minor significance changes for some associations that were borderline significant in Model 1: between age and physical fatigue, education and anxious symptoms and living situation and mental fatigue.

**Supplementary Table S5:** Moderation Analysis results to explore effects of COVID-19 living situation (self-isolating vs not self-isolating) on all associations between independent and dependent variables

|  | **Pain (MPQ)** | | | **Pain (VAS rating)** | | | **Physical Fatigue** | | | **Mental Fatigue** | | | **General Fatigue** | | | **Anxious Symptoms** | | | **Depressive Symptoms** | | | **Vitality** | | |
| --- | --- | --- | --- | --- | --- | --- | --- | --- | --- | --- | --- | --- | --- | --- | --- | --- | --- | --- | --- | --- | --- | --- | --- | --- |
|  | β | 95 % CI | | β | 95 % CI | | β | 95 % CI | | β | 95 % CI | | β | 95 % CI | | β | 95 % CI | | β | 95 % CI | | β | 95 % CI | |
|  |  | Lower limit | Upper limit |  | Lower limit | Upper limit |  | Lower limit | Upper limit |  | Lower limit | Upper limit |  | Lower limit | Upper limit |  | Lower limit | Upper limit |  | Lower limit | Upper limit |  | Lower limit | Upper limit |
| **LPA** | -.06 | -0.24 | 0.12 | -.03 | -0.21 | 0.15 | -.02 | -0.20 | 0.17 | -.22* | -0.43 | -0.01 | -.15 | -0.35 | 0.04 | -.11 | -0.32 | 0.09 | -.10 | -0.30 | 0.10 | .28* | 0.08 | 0.48 |
| **Walking** | -.03 | -0.21 | 0.15 | .01 | -0.17 | 0.20 | .20* | 0.01 | 0.39 | .19 | -0.02 | 0.41 | .06 | -0.14 | 0.26 | .04 | -0.17 | 0.25 | .05 | -0.15 | 0.26 | -.02 | -0.23 | 0.19 |
| **Exercise** | -.04 | -0.24 | 0.15 | .15 | -0.05 | 0.35 | .01 | -0.19 | 0.21 | .05 | -0.18 | 0.28 | -.05 | -0.26 | 0.16 | -.08 | -0.30 | 0.15 | -.06 | -0.28 | 0.16 | -.01 | -0.23 | 0.22 |
| **Sedentary Time** | -.05 | -0.24 | 0.15 | -.12 | -0.32 | 0.08 | .04 | -0.15 | 0.24 | .21 | -0.02 | 0.44 | .05 | -0.16 | 0.26 | -.14 | -0.36 | 0.08 | .00 | -0.22 | 0.22 | -.09 | -0.31 | 0.14 |

Note: Significant associations (=*) between LPA, walking, exercise and sedentary time (independent variables) with indicators of mental health and wellbeing (dependent variables) were derived using bootstrapped 95% CI

Moderation Analysis: COVID-19 living situation (self-isolating vs not self-isolating) was added as a moderator variable to Model 2 regressions (LPA, walking, exercise or ST, adjusted for all covariates), using the PROCESS model in SPSS, to explore if COVID-19 living situation moderated the associations between the independent and dependent variables, i.e., if self-isolation status affected associations between activity behaviour and indicator of mental health and psychological wellbeing.

Results are reported using Model 2 regressions (adjusted for covariates: age, gender, living situation, education, general COVID-19 concern and functional disability). β coefficients represent the degree of change in the outcome dependant variable for every 1 unit change of the independent variable. β coefficients and 95% CIs were calculated using z scores of all independent and dependant variables in order to standardise output values.

LPA= light non-exercise physical activity, MPQ= McGill Pain Questionnaire, VAS= Visual Analogue Scale, β= standardised beta coefficient, 95% CI= 95% confidence intervals.
